# Gold Nanoparticles for Diagnostics: Advances towards Points of Care

**DOI:** 10.3390/diagnostics6040043

**Published:** 2016-11-22

**Authors:** Mílton Cordeiro, Fábio Ferreira Carlos, Pedro Pedrosa, António Lopez, Pedro Viana Baptista

**Affiliations:** 1 UCIBIO, Departamento de Ciências da Vida, Faculdade de Ciências e Tecnologia, Universidade Nova de Lisboa, Campus da Caparica, 2829-516 Caparica, Portugal; m.cordeiro@campus.fct.unl.pt (M.C.); fa.carlos@campus.fct.unl.pt (F.F.C.); pm.pedrosa@campus.fct.unl.pt (P.P.); a.lopez@campus.fct.unl.pt (A.L.); 2 Rede de Química e Tecnologia (REQUIMTE), Departamento de Química, Faculdade de Ciências e Tecnologia, Universidade Nova de Lisboa, Campus da Caparica, 2829-516 Caparica, Portugal

**Keywords:** gold nanoparticles, diagnosis, point-of-need, biomolecular sensing

## Abstract

The remarkable physicochemical properties of gold nanoparticles (AuNPs) have prompted developments in the exploration of biomolecular interactions with AuNP-containing systems, in particular for biomedical applications in diagnostics. These systems show great promise in improving sensitivity, ease of operation and portability. Despite this endeavor, most platforms have yet to reach maturity and make their way into clinics or points of care (POC). Here, we present an overview of emerging and available molecular diagnostics using AuNPs for biomedical sensing that are currently being translated to the clinical setting.

## 1. Introduction

Precise and accurate diagnosis of human related diseases (i.e., genetic disorders, pathogen infection, etc.) is of paramount importance for health care in both developed and developing countries. It ensures that patients have access to the most favorable therapeutic agents in the shortest time span, leading to better prognosis. This translates to significant reductions in the financial burden for health care systems [1]. In developed countries, diagnostics is usually performed at centralized laboratories by specialized personnel. In developing countries, where these infrastructures usually lack the appropriate equipment and/or personnel, accurate diagnosis may be cost-prohibitive and therefore inaccessible [2]. To overcome these bottlenecks, technologies that allow diagnosis at the site of care—point-of-care testing (POCT)—are of extreme importance, allowing for a reduction in sample transportation and processing, use at the point of need, and more importantly, a shorter time between diagnosis and appropriate therapeutic intervention.

The World Health Organization set criteria for POCT, summarized in the acronym “ASSURED”—affordable, sensitive, specific, user-friendly, rapid/robust, equipment-free or minimal and deliverable to those with the greatest need. By fulfilling most of these requirements, POCT should bring not only traditional centralized laboratory-based testing (sensitive and specific) closer to both patients and doctors (user-friendly), but also be suitable for low-income countries where the lack of healthcare facilities is a reality. POCT devices may be grouped into two categories: (i) miniaturized devices with automate sample preparation, analysis and detection to be used on a hand-held basis and (ii) robust devices that allow increased sensitivity for a wide spectrum of analytes to be used at the benchtop [3,4]. The biggest challenge is to make these tests and/or devices consumable and available at a low-cost with reliable results.

Recent developments in nanotechnology have put forward a wide range of nanosensing platforms with unique properties that are revolutionizing molecular diagnostics [5]. Here, a focus will be given to gold nanoparticle (AuNP)-based platforms that are suitable for POCT, stating the physical-chemical principals and biomolecular recognition used in these platforms, promising state of the art proof of concepts that may be suitable for POCT and finally some examples of current status of the market in this field.

### 1.1. Gold Nanoparticles (AuNPs)—Properties and Sensing Applications

#### 1.1.1. Localized Surface Plasmon Resonance (LSPR)

**Figure 1:**
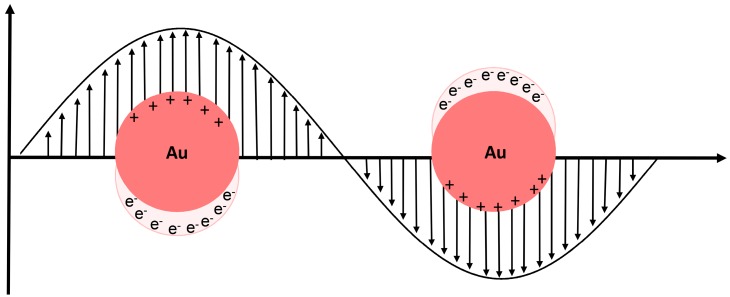
Schematic representation of metal nanoparticles in localized surface plasmon resonance (LSPR). Interaction of the electromagnetic waves with the metal nanoparticle (NP) surface electrons (e^−^) induces a surface plasmon resonance.

Colloidal AuNPs have been extensively used for diagnostic apparatus, due to their optical properties, ease of synthesis and surface functionalization. A very important optical property of AuNPs is the presence of a localized surface plasmon resonance (LSPR), which can be described as the collective oscillation of the conductive electrons of the gold atoms that is triggered through the interaction with an incident electromagnetic wave (i.e., light)—Figure 1. This collective oscillation generates a polarization of the AuNP, inducing the formation of dipole moments that leads to the extinction of the electromagnetic wave with the appropriate frequency [6].

LSPR is highly dependent on size, shape, composition, inter-particle distance and dielectric surroundings. Therefore, it can be finely-tuned through the manipulation of these variable using various synthesis routes (for a review see [7] and references therein) or dispersion media [8]. LSPR results from the absorption and the scattering of the AuNPs, and the relative weight of each to the overall LSPR can be controlled through the size and shape of the AuNP. For instance, small and regular AuNPs tend to favor the absorption component while larger and more irregular AuNPs tend to favor the scattering component [8]. The LSPR extinction of incident light is so strong that the extinction coefficient associated with AuNP is usually three orders of magnitude higher than in conventional dyes [9], making them suitable agents for optical sensing applications. Usually, a solution of AuNP will present a deep red color due to the LSPR (absorption in the green). As the diameter increases, the extinction band red-shifts, yielding a bluish/purple solution. This red-shift of the LSPR is also observable when monodispersed particles couple their dipole moment either through proximity effects (aggregation) or by a designed controlled interaction. This high extinction coefficient allows for higher sensitivity and lower limits of detections (LOD) [10], while the red-to-blue color shift (spectral red shift) is a convenient output signal that has been exploited for the development of several visual colorimetric biosensors. The red-shift property was applied in the development of an immunoassay for the detection of *Mycoplasma pneumonia*, where the alkaline peroxidase label of the secondary antibody catalyzes a series of chemical reactions that leads to the formation of copper (I) that in turn triggers the interaction of azido- and alkyne-functionalized AuNPs, shifting the solution from red to blue [11]. A similar approach was used for the development of a plasmonic variation of the widely used enzyme linked immune sorbent assay (ELISA) [12], Tfor the detection of prostate specific antigen (PSA) and a HIV-associated protein, p24, where the secondary antibody was labeled with an enzyme that generated a compound that induced the formation of AuNPs [13]. The red-shift property based on dipole coupling has been used for the detection of wide range of analytes from nucleic acids to small molecules such as cocaine [14].

#### 1.1.2. Fluorescence Modulation

Fluorescence is an optical phenomenon where the absorption of a photon is followed by the emission of a lower frequency photon. The energy difference between the absorbed and emitted photon is a result of the vibrational relaxation of the excited-state molecule, and it is responsible for the Stokes shift [15]. Fluorescence has been widely used in diagnostics procedures, such as in quantitative real-time polymerase chain reaction (qPCR) for the diagnosis of chronic myeloid leukemia [16], DNA sequencing [17]], flow cytometry [18], fluorescence microscopy [19] and in vivo imaging [20].

**Figure 2:**
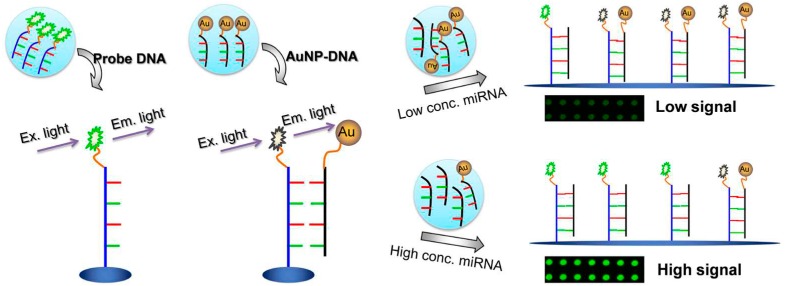
Schematic representation of miRNA-205 competitive detection based on fluorescence quenching of gold nanoparticles (AuNPs). Adapted from [28].

A particular fluorophore’s emission may be modulated by the proximity to a AuNP, due to interaction between the fluorophore and the LSPR field [21]. It has been reported that AuNPs can interfere with the radiative and non-radiative pathways of excited state fluorophore deactivation, and therefore, are able to induce both a suppression (quenching) or enhancement of the emitted light. The equilibrium between the radiative and non-radiative constants dictates whether quenching or enhancement occurs [22]. As such, AuNP LSPR needs to be finely tuned and compatible with the photochemical properties of the fluorophore for the interaction to occur. Both effects can be used for the development of biosensors, considering that enhanced emission can be used for higher signal-to-noise ratios and the quenching effect has been used for the development of “on/off” sensors. These are sensors whose emission output is dependent on the presence/absence of the analyte, whereby: (1) the presence/binding of the target analyte allows for fluorescence recovery, for example, through the removal of the fluorophore from the surface of AuNPs [23,24]; (2) the presence of target analyte induces the approximation of the fluorophores and the AuNP surface, inducing the quenching of fluorescence emission [25,26,27]. For example, a competitive assay for the detection of miRNA-205 was developed, where the absence of target sequence keeps the fluorophore in the vicinity of an AuNP due to complementary strands, causing emission suppression. Upon competitive binding to the target sequence, the AuNP bearing the complementary strand is displaced and the fluorophore emission is recovered [28] (Figure 2). Most of the developments in this field enhance the applicability of using fluorescence and AuNP for sensing application, with the majority using the “on/off” approach due to the quenching effect of AuNP.

#### 1.1.3. Surface-Enhanced Raman Scattering (SERS)

Raman scattering is a vibrational spectroscopic technique relying on the inelastic collision between an incoming source of light and an analyte of interest. This inelastic collision is responsible for the scatter of a lower energy radiation that serves as a fingerprint and provides information regarding structure, interaction or environment of the analyte [29]. However, the probability of Raman scattering is very low, hindering its application as a routine platform for diagnosis. A 10^6^ amplification increase of the Raman signal is possible by means of noble metal surfaces and structures, such as gold and silver. This signal amplification is known as surface-enhanced Raman scattering (SERS) and allows the development of sensitive procedures for characterization of target molecules. This signal amplification can occur, either by interaction of the incident and scattered photons with the SPR of the metal surface, or a charge transfer between the metal surface and the target molecule [30]. This phenomenon has been applied in the detection of nucleic acids, antibodies, proteins and other biological molecules [31,32,33], and small molecules such as glucose [34].

#### 1.1.4. Electrochemistry

The high surface-to-volume, high conductivity and catalytic properties of AuNPs make them suitable platforms for the development of electrochemical sensors for a wide array of biomolecules, from the detection DNA sequences and proteins with clinical relevance [35,36] to small molecules such as estradiol [37]. The electronic properties of AuNPs can be controlled through size, particle separation and surface modification [38]. AuNPs are capable of decreasing the redox over-potential of redox reactions [39] [and sustain the reversibility of redox reactions [40,41]. As such, AuNPs have been extensively used as electrochemical labels and carriers of biomolecules for detection of clinical relevant analytes, such as DNA [35], cancer-associated proteins [42] and circulating cancer cells [43]. Additionally, AuNPs have been used to modify the electrode surface in order to increase the redox surface area, allow the immobilization of biomolecules and improve direct electron transfer which allows for electrochemical signal amplification [5].

AuNPs are also suitable for other diagnostic modalities, such as photoacoustic and X-ray imaging due to their higher contrast capability compared to standard compounds [44,45]. However, the need for bulky equipment and specialized personnel hampers their implementation at points of care (POC) in the current state of technology.

## 2. General Principles of AuNP-Based Biomolecular Recognition

A key aspect in the development of a biosensor is its ability to specifically detect the target molecule from a pool of non- or closely-related molecules. As such, the choice of biorecognition element—bio receptor—is of vital importance. Given that the surface chemistry of AuNPs can be tailored through the incorporation of different functional moieties [46], it is possible to modulate the affinity of an AuNP towards a wide range of analytes. There are three main methods for the surface modification/functionalization of AuNPs: (1) Adsorption-based, where the interaction between the ligand and the AuNP surface is held by electrostatic or hydrophobic interaction; (2) Covalent bonding, where the ligand is linked to the AuNP surface through a thiol group, either direct conjugation of sulfur containing molecule or through a bi-functional linker (a thiol group at one extremity that binds to the AuNP and another functional group at the other extremity, where other biomolecules can be attached); and (3) Affinity-based, where the AuNP surface is functionalized with moieties that provide affinity sites for the coupling of biomolecules (please refer to reference [47] for a detailed review of common functionalization strategies). Due to the ease of functionalization coupled to the aforementioned properties, AuNPs are a very versatile scaffold for the development of sensing platforms. Herein, the most prevalently used bioreceptors that allow AuNPs to have specific biomolecular recognition abilities will be described.

### 2.1. Nucleic Acids Sensing

Nucleic acids (DNA or RNA) are widely used in sequence dependent interactions of many hybridization assays, such as fluorescence in situ hybridization (FISH) [48,49], DNA amplification techniques, such as PCR [50], loop-mediated isothermal amplification (LAMP) [51], hybridization chain reactions (HCR) [52] and microarray technology [53]. Also, there are chemical analogues of nucleic acids that can be used as bioreceptors, such as locked nucleic acids (LNA) and peptide nucleic acids (PNA). For LNA, the ribose of the nucleotide contains a methylene bridge between the 4′ carbon and 2′ oxigen, yielding in a less flexible conformation due to a more pronounced base stacking [54], which leads to higher affinity towards the complementary strand as demonstrated by higher melting point of the duplexes [55,56]. For PNA, the phosphate backbone is replaced with *N*-(2-aminoethyl)-glycine molecules linked by peptide bonds, which removes the electrostatic repulsion between strands, allowing for more efficient hybridization. PNAs and LNAs are also more resistant to nucleases [57,58], adding to the stability of the bioreceptor in complex media. These characteristics make both LNA and PNA suitable for biosensing.

Mirkin et al. first described the use of thiolated oligonucleotides (short single strand DNA, ssDNA) as a capping agent for AuNP in 1996 [59]. This ssDNA-modified AuNP, through a precise temperature control, was able to discriminate between a base pair mismatch from a fully complementary target [60]. Since then, there have been several studies demonstrating the vast capability of AuNPs functionalized with short nucleic acid sequences to sense a wide range of clinically relevant nucleic acid sequences (as exemplified in [61,62,63,64,65]).

**Figure 3:**
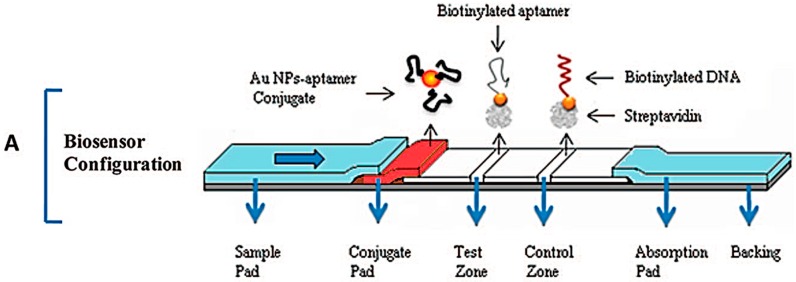

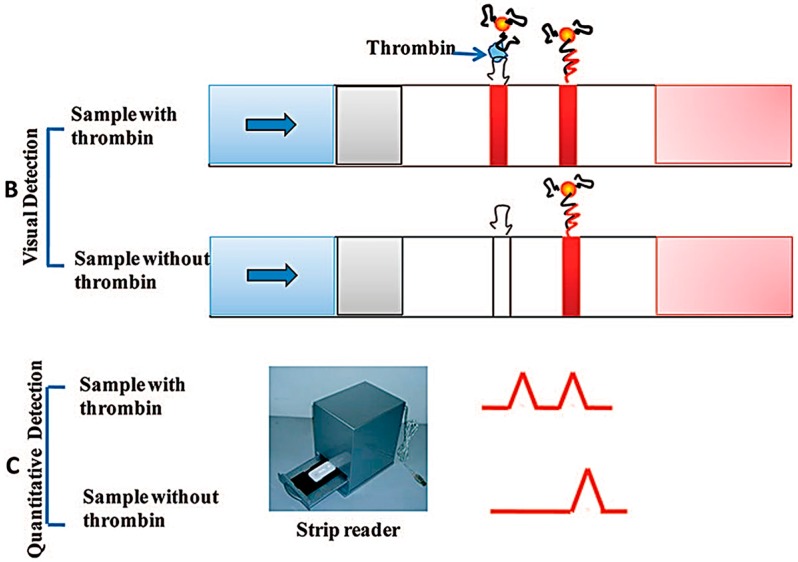
Schematic illustration of the configuration and measurement principle of the aptamer-based strip biosensor: (**A**) configuration of the biosensor; (**B**) the principle of visual detection in the presence and absence of thrombin; (**C**) quantitative detection with a portable strip reader. Adapted from [70].

Nucleic acids as bioreceptors are not limited to hybridization based detections. Aptamers are a special class of nucleic acids that have affinity towards a wide range of analytes. These are short sequences of nucleic acids which have a secondary structure with a structural affinity towards a given analyte or which acquire a three-dimensional structure upon analyte binding [66]. These interactions are ruled by a combination of pi bond stacking, London dispersion forces and hydrogen bonding [67,68]. Some aptamers have been developed to bind to wide range of analytes such as metal ions [69]], proteins [70,71] and whole cells [72]. Aptamers are generated through a process denominated systematic evolution of ligands by exponential enrichment (SELEX) [73]. Briefly, a large library of short oligonucleotides is exposed to a target molecule, such as proteins or organic compounds, and the oligonucleotides that do not interact with the target molecule are removed. The procedure is repeated multiple times until a high specificity and affinity interaction between oligonucleotides and target molecule is obtained, with dissociation constants ranging from picomolar to nanomolar [74]. As such, they are versatile as bio-recognition molecules for sensing applications due to their antibody-like specificity coupled with their ease of synthesis, higher thermal stability, cost-effective production, wide range of analytes that can be targeted, lower batch to batch variation and simple modification with different chemical moieties [75]. A lateral flow strip (LFS) for thrombin was developed using aptamer-functionalized AuNPs (apt-AuNP). The analytical performance of the apt-AuNP LFS was superior to the antibody equivalent and allowed the unequivocal visual detection of thrombin. By using a strip reader, more precise detection was possible, enabling analyte quantification [70]—see Figure 3.

### 2.2. Protein Sensing

Proteins are another class of biopolymers that can be used as bioreceptors in biosensing platforms. Antibodies (Ab) are one of the most used protein bioreceptors for biosensing due their specificity towards their respective antigen. The specificity between the Ab paratope and antigen epitope interaction has been applied in several immunoassays for clinical diagnosis through the well-established ELISA [76] and their use has been extended to POC-suitable testing such as lateral flow assays (LFA), i.e., pregnancy tests [77].

Peptides and enzymes are also versatile bioreceptors for AuNP-based sensing as they can be functionalized to the surface of AuNP without losing their biorecognition capabilities. For instance, the high local concentration of immobilized enzymes at the surface of AuNP coupled to their catalytic specificity can be used for signal enhancement in LFA platforms, due to a higher local concentration of the colored product (revelator). This principle was applied for the detection of human IgG in an LFA format [78]. Peptides on the other hand, can serve as binding partners of an interaction event or be a substrate for specific reactions. There have been reports of peptide functionalized AuNPs for the detection of metal ions [79,80] as well as for the determination of kinase activity [81].

## 3. General Overview of Applications

### 3.1. Lateral Flow Assays (LFAs)

LFAs use widespread technology suitable for POC diagnostics [5,82,83,84,85] with user-friendly handling, fast turnaround time to result, low-cost, acceptable specificity and extended shelf life [86]. A 2010 study revealed that LFAs represented 50% and 40% of the overall rapid test market in the US and Europe, respectively [87]. The capillary action of an LFA is able to transfer biological fluids, including blood or serum, not requiring an external power supply [82,85]. Since the first human pregnancy strip test, LFA technology has been steadily taking an increasing market share of POCT, whose applications include infectious diseases such as HIV, malaria, tuberculosis, influenza, and others [83,84].

**Figure 4:**
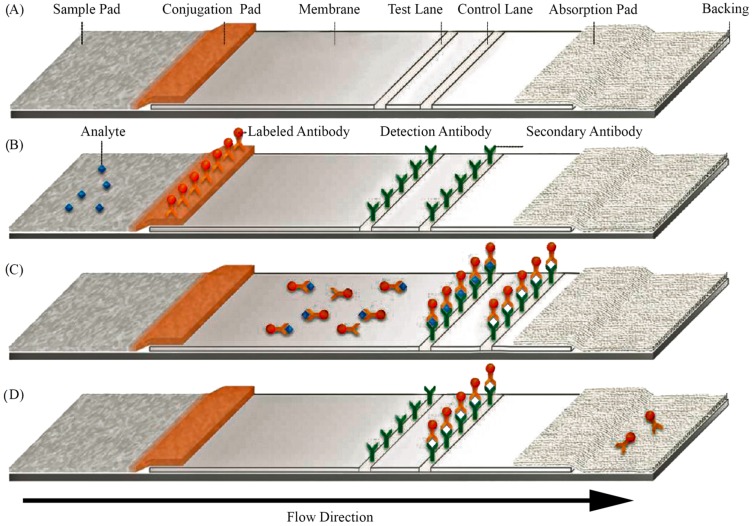
Overview of lateral flow assay (LFA) principle. (**A**) Physical components of a conventional LFA system; (**B**) Biological components present in a conventional LFA; (**C**) Upon addition of a positive sample, the labeled antibody interacts with the analyte and migrates by capillary action to the test lane, where the complex analyte-antibody is immobilized through the detection antibody. The control lane also immobilizes the labeled antibody; (**D**) In the absence of the target, the labeled antibody does not interact with the detection antibody and is immobilized in the control lane, indicating negative detection of the analyte. Adapted from [90].

Generally, LFAs are based on a sandwich working principle, where the target analyte bridges the strip-immobilized bioreceptor with the revelator molecule/construct: the sample is inserted on a sample pad, then migrates through to the conjugation pad where the first recognition agent is present and interacts with the analyte. The resulting complex further migrates to the reaction layer—a hydrophobic nitrocellulose or cellulose acetate membrane—where an immobilized probe captures the labelled conjugate. A control layer is usually also present, capturing the first recognition element in order to assess correct functionality of LFAs—see Figure 4. Results are interpreted either visually or using a strip reader for a more precise determination [83,84,85,86].

Despite providing reliable results with acceptable sensitivity, a lack of robustness and reproducibility are the main drawbacks of LFAs. Incorporation of AuNPs as signal transduction moiety has provided increased sensitivity and sensitivity [83,88]. AuNP-based LFAs can be improved by the deposition of hydrophobic barriers (e.g., wax printing) on the detection pad of the LFA. These barriers act as obstacles, delaying the regular capillary flow to increase the binding time between the analyte and the bioreceptor, increasing the number of probe-target complexes. These hydrophobic depositions can be easily extended to other LFA designs, and by using different patterns, for instance, their versatility can be extended to POC applications [86]. The substrate of the LFA can also be optimized, using cotton for example, a flexible, widely available and easy-to-handle material, which requires a lower sample amount and adds increased robustness of the strip, surpassing the drawbacks of the common LFA [89].

**Figure 5:**
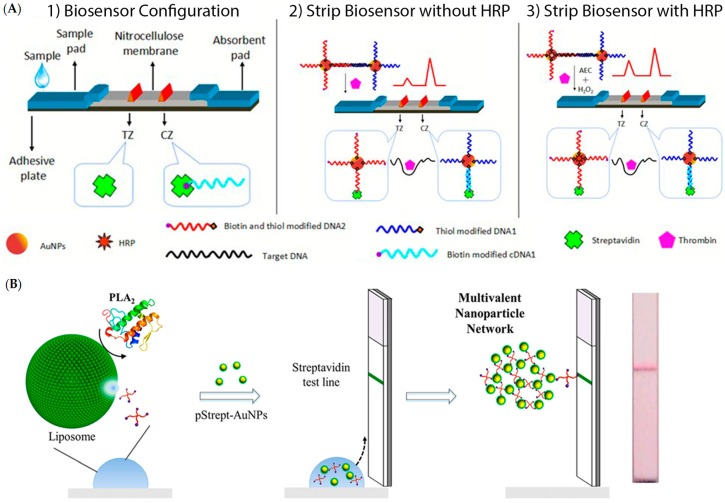
Examples of signal amplification methodologies for AuNP-based lateral flow strips. (**A**) horseradish perioxidase (HRP)-functionalized AuNPs allow signal amplification for the aptamer-based detection of thrombin (TZ: test zone; CZ: control zone); (**B**) Network of Streptavidin-functionalized AuNPs allow for visual detection of AuNP. Adapted from [91] and [92].

Using a cotton LFA test with AuNPs, it was possible to detect human ferritin, a biomarker for lung cancer, with an LOD of 10 ng/mL, which is sensitive enough for clinical diagnosis. Signal amplification strategies can also be applied in an LFA format. The use of LFAs for the detection of thrombin has been reported—see Figure 5 A1, where a thrombin aptamer acts as a crosslinker between two populations of AuNPs [91]. Upon thrombin binding, the AuNP complex is disrupted and each population hybridizes either to the test or control lane—see Figure 5 A2. The AuNPs binding to the test lane are labelled with horseradish peroxidase (HRP) which generates a colored product that allows for a higher output signal—see Figure 5 A3. The LOD was set at 4.9 pM and the assay can be performed in 12 min using 6.4 pM of the target molecule without instrumentation.

In another approach, signal-amplification LFA was used for detection of human phospholipase A2 (PLA2) [92]. Biotinylated-polyethylenoglicol (Bt-PEG)-loaded liposomes are disrupted in the presence of the target analyte—See Figure 5 B. This disruption leads to the leakage of the Bt-PEG that acts as a crosslinker for streptavidin-coated AuNPs. The formed AuNP net is then immobilized in the streptavidin coated test lane, allowing visual detection of 1 nM of PLA_2_ under 10 min. However, the absence of a control lane may hinder the real applicability of this test due to the inability to guarantee the quality of the signal generated.

An LFA for the detection of antibodies anti-*Treponema pallidum* (Tp), the etiologic agent of syphilis [93], was developed using iron oxide core-shell magnetic AuNPs with a polyacrylic acid (PAA) coating [94]. The polymeric coating provides a hydrophilic nature to the AuNP surface while containing chemical moieties to covalently attach bioreceptors. The iron oxide core allows for magnetic purification of the constructs in the preparation phase of the LFA, and the optical properties of the gold shell allowed for visual detection of anti-Tp antibodies from sera, with an LOD of 1 national clinical unit per mL.

**Figure 6:**
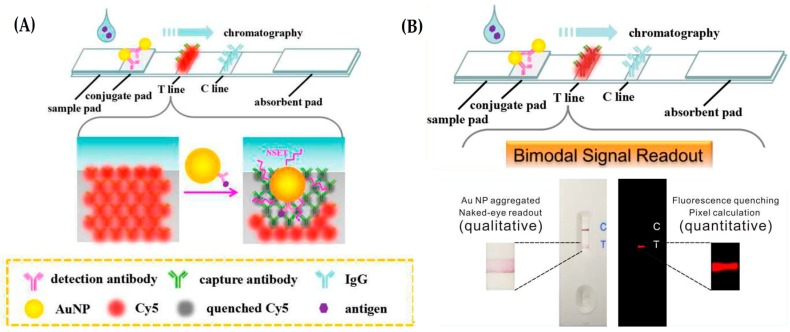
Dual Signal output lateral flow assay for the detection of carcinoembryonic antigen. (**A**) Mechanism of fluorescence quenching signal output for antigen quantification; (**B**) Visual (qualitative) and fluorescence output (quantitative) for clinical detection. Adapted from [95].

The versatility of AuNPs for the development of an LFA-based platform was further demonstrated in the qualitative and quantitative detection of carcinoembryonic antigen (CEA) from human sera [95]. Here, fluorophore-labeled Abs are immobilized on the test lane, and upon positive molecular recognition, the accumulation of the AuNP leads to the generation of a positive red line (due to the AuNP LSPR)—See Figure 6. This set-up allows for a bimodal signal readout; visually for the red line or by pixel quantification of the emission of the fluorophore. For the quantitative approach, the quenching efficiency of the fluorophore label of the immobilized Ab is used and allows for an LOD of 5.89 pg/mL. This assay can be observed visually in under 10 min.

### 3.2. Microfluidics

Microfluidic technology is based on the design and manufacturing of systems in which low volumes of fluids are used. It has emerged in the interface of several fields (engineering, physics, chemistry, nanotechnology, and biotechnology) aiming at precisely controlling and manipulating fluids that are restricted in small compartments. These fluids can be moved, mixed or separated simply by using capillary forces or by using active components such as micropumps or microvalves. Micropumps supply continuous fluid to the system while microvalves define the direction flow of liquids [96]. Microfluidic technology is excellent for POC tests due to its higher surface to volume ratio, fast rate of heat and mass transfer and reduced volume of sample (nano or even picoliters) [97]. The use of microchannels allows for high handling precision of reagents while reducing costs and time of analysis while integrating all components of molecular detection in a single platform, namely: purification, amplification, and detection [83]. However, as the complexity of fluid circuits and microfabricated valves or pumps increases, so do the costs and the need for expensive and large external equipment, with neither miniaturized nor portable alternatives. An ideal microfluidic readout system should be fast, portable, sensitive and quantitative, while allowing the detection of a wide range of targets [83,97]. AuNPs have been implemented in microfluidic systems for biomolecular detection of nucleic acids, proteins, and small molecules [98,99] improving the sensitivity and specificity of the assays, and expanding their range of detection.

Several microfluidic devices have been developed for the detection of pathogen biomarkers and use in food and water sources in this context. Rudi Liu et al. developed a portable readout V-Chip, suitable for POCT of Ochratoxin A with an LOD of 1.27 nM (0.51 ppb) in biological samples [100]. Pires and Dong have described a microfluidic biosensor for detection of *Legionella pneumophila* in water with a resolution of 4 × 10^4^ cells/mL representing a 25-fold improvement over chemiluminescent detection devices [101]. Ölcer et al developed a microfluidic- and nanoparticle-based amperometric biosensor for nucleic acid detection of cyanobacteria. The biochip was fabricated on a silicon dioxide wafer that consists of two gold electrodes, each with a reference/counter electrode and three working electrodes, with each set sharing a gold counter and a quasi-reference electrode. A sensor cassette of poly(methyl methacrylate) and double- sided sticky tape was fabricated forming a microfluidic channel on the electrode array. They also set up a potentiostat, a syringe pump, an injection valve and a sensor chip docking station for the detection assays with a flow rate of 50 mL/min. The AuNPs were functionalized with an HRP-labeled ssDNA oligomer complementary to a cyanobacteria DNA sequence, which, after hybridization, formed a DNA sandwich with an immobilized probe. An electroactive substrate is then added to the chip for electrochemical readout. The LOD of 6 pM was obtained with an assay that takes about 75 min to complete [102].

Other platforms for the detection of metabolites of clinical relevance have been reported, such as cortisol [98], glucose [99] and dopamine [103]. Additionally, the use of these platforms for DNA molecular diagnostics of human pathologies has been proposed with the potential for discrimination of point mutations [104,105]. However, fabrication of low-cost microfluidic devices is challenging due to conventional fabrication techniques involved i.e., photolithography, etching, electron beam lithography, printing, and molding, involving costly technology and expert personnel. Thus, paper-based microfluidics is an emerging field mainly due to its fabrication simplicity. Mostly using cellulose of silica based matrixes as substrate, a few detection techniques have been integrated with microfluidic devices, using colorimetry, fluorescence, electrochemical and SERS readout. Among them, Saha and Jana implement this paper concept with silver/gold core shell nanoparticles for protein detection at picomolar concentration as a proof of concept. This approach is based upon aggregation of nanoparticles functionalized with a Raman reporter in the presence of proteins. Both particles and the target are placed in two different spots of the paper and migrate to the reaction zone, where particle aggregation and electromagnetic hot spot generation results in a reproducible SERS signal. Although it still lacks optimization, this approach has the potential to become a SERS-based POC tool [106].

### 3.3. Screen Printed Electrodes

**Figure 7:**
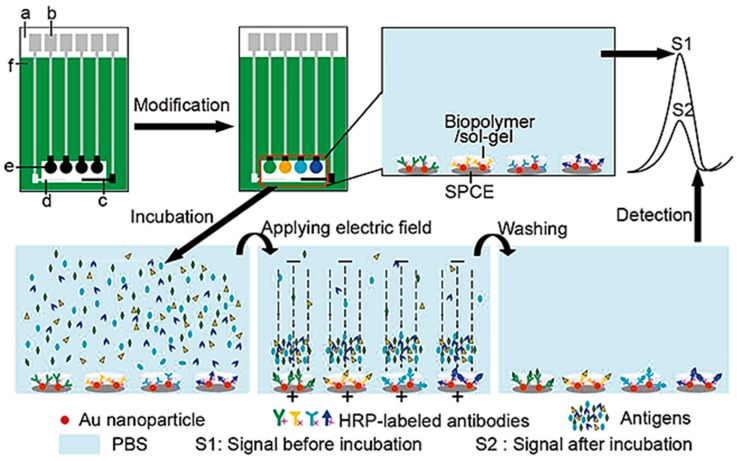
Schematic representation of an electrochemical multiplexed immunoassay with an electric field-driven incubation process. (a) nylon sheet; (b) silver ink; (c) graphite auxiliary electrode; (d) Ag/AgCl reference electrode; (e) graphite working electrode; and (f) insulating dielectric. Adapted from [112].

Screen-printing technology has emerged as the method of choice for large scale fabrication of POC sensors. Screen-printed electrodes (SPEs) are widely used in mass production of reproducible and inexpensive electrochemical sensors, creating disposable, low cost, real-time biosensing devices [5]. SPEs are generally composed by working, counter, and reference electrodes. At the working electrode the electrochemical reactions occur, while both the reference and counter electrodes are employed to complete the electronic circuit. Several working electrodes can be used in the same chip, allowing the detection of several analytes in the same sample, such as cancer biomarkers. Different inks and substrates have been reported in SPEs such as carbon, ceramics, plastic, fiber glass or gold, iron and silver. The ink formulations can vary in type, size or loading of particles which influences the electron transfer reactivity and changes the biosensor performance, defining its selectivity and sensitivity. Carbon-based inks are particularly desirable due to their relatively low-cost, low-background currents, chemical inertness and broad potential windows [107]. Gold inks have gained some interest due to their ease of functionalization with biomolecules through thiol bonding, despite their higher cost [108,109]. SPEs are highly versatile, with multiple possible combinations of inks and functionalization (like polymers, enzymes or DNA) [107,109] and they are highly sensitive to current variation, allowing the use of sample volumes in the range of microliters. In the last decade, nanomaterials have been incorporated in the fabrication of SPEs such as nanoparticles, nanowires, carbon nanotubes and graphene [5,110,111]. These materials assist the immobilization of biological targets and change the charge transfer rate on the working electrode surface. AuNPs have been described in these systems, generally deposited on the working electrode or mixed with the sample in a sandwich approach. Jie Wu et al. reported the use AuNPs impregnated in a biopolymer/sol-gel matrix deposited in an SPCE for the simultaneous detection of four cancer biomarkers using clinical samples [112]—see Figure 7. By using four working electrodes with AuNPs functionalized with different antibodies, the authors were able to separately detect in each electrode a specific biomarker, resulting in a pattern for each sample. The positivity detection rate of panels of tumor markers was 95.5% for 95 cases of cancer-positive sera and with a shelf life of at least 35 days [112]. Duangkaew et al. also describe the use of functionalized AuNPs in an SPCE, for cancer detection [113]. They used a sandwich approach in which a cervical cancer biomarker (GST-p16) served as a linker, binding antibodies in the carbon electrode and in the AuNPs. The sample was then silver-enhanced, increasing the current signal due to the proximity of the nanoparticles. After analysis of 20 clinical samples, the LOD was set at 1.3 ng/mL for GST-p16 protein which is equivalent to 28 cells for HeLa cervical cancer cells [113]. Disposable biosensors employing screen-printing technology have already proven their commercial success for diabetes management due to the multi-billion-dollar glucose monitoring market [114]. Considerable advancement is still needed towards integration of SPEs and fluid-handling and/or sample-processing tools to ensure portable POC devices for cancer and pathogen diagnostics [35].

SPEs are suitable to make miniature devices capable of giving reproducible results with high sensitivity in biochemical detection [5,111]. In addition, application of SPE arrays provides the benefits of speed and the possibility to carry out calibration and analysis of several unknown samples.

### 3.4. Smartphone Assisted Readout

**Figure 8:**
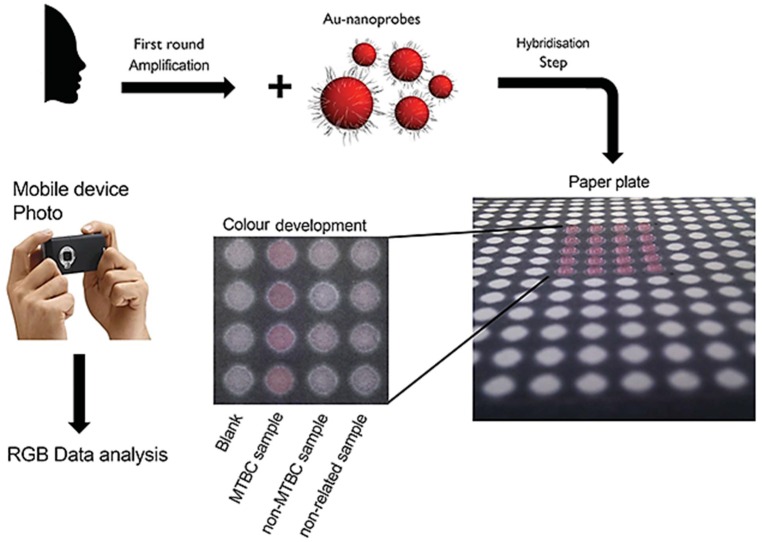
Au-Nanoprobe strategy for the detection of MTBC members. Schematic representation of the detection with gold nanoprobes. The colorimetric assay consists of visual comparisons of test solutions after salt induced Au-nanoprobe aggregation on a MgCl_2_ impregnated paper plate: MTBC Au-nanoprobe alone (blank); MTBC Au-nanoprobe in the presence of MTBC sample (*M. tuberculosis*); MTBC Au-nanoprobe in the presence of a non-MTBC sample; and MTBC Au-nanoprobe in the presence of a non-complementary sample (non-related). After color development a photo of the paper plate was captured and RGB image analysis was performed. Adapted from [61].

The use of modern consumable devices with imaging capabilities (e.g., cameras and smartphones) to acquire and analyze optical readouts at POC (e.g., colorimetric changes of AuNPs [61]) provides reliable qualitative results (operator independent—see Figure 8) and also accurate quantitative results [115]. The exploitation of smartphone-, tablet- and portable camera-properties offers a helpful starting point for diagnostic platforms in low or absent-resource areas. In remote diagnostics, results may be easily transmitted to a central laboratory for in depth analysis by experts [116]. In addition, all information may be centralized, stored and organized for posterior analysis (i.e., demographic data, prevalence, incidence. etc.) [117], which is of extreme relevance for epidemiological data gathered in isolate populations of remote areas in developing countries [118,119]. Tests for the detection of heavy metals, malaria, tuberculosis and for the quantification of vitamins (such as B_12_ and D) and glucose have been reported by taking advantage of approaches relying on smartphones [61,117,119,120,121,122,123].

## 4. AuNPs Based Systems Established at POC

### Achievements and Challenges to Overcome

The advantages of integrating AuNPs into portable, low-cost and miniaturized platforms may be summarized in three points: (i) ease of AuNP synthesis with acceptable reproducibility and homogeneity; (ii) AuNP surface proprieties can be finely tuned to provide different sizes and shapes; and (iii) ease of surface modification and exceptionally stability. The use of AuNPs in colorimetric detection approaches has been widely explored and, due to the simplicity and portability, is the most capable for implementation in POC strategies [47,124,125]. POC based on AuNPs still are in its primary stages (prototype) of development and use at bedsides, physician's offices, healthcare facilities, and in remote care settings is so far limited [126].

POC diagnostics is an extremely attractive and growing market, with a value of €15.5 billion in 2013 and a compound annual growth rate (CAGR) of 4.5% estimated for 2018 (i.e., an estimated market value of €19.3 billion in 2018) [127]. Translation of the promising diagnostics platforms based on AuNPs to a clinical environment requires appropriate guidelines. Such guidelines are still not clear, since most countries have still to regulate standardization, production and operation of such nano-based materials. In limited or non-existing healthcare service areas, where the access to primary-care facilities is especially difficult, POC approaches may constitute a valuable asset as a primary screening tool [128].

One of the most relevant and effective strategies currently being used for the development of POC AuNP-based detection systems are immunological LFA assays [129]. Simple instrumentation, low-cost fabrication, portability and long shelf-life [82] are some of most outstanding characteristics related to LFA tests that are being exploited in AuNP-POC-related products already available on the market [130]. Some LFA AuNP-based tests available on the market have already been compared with standard conventional techniques (ELISA), overcoming them in performance. For example, the five LFA AuNPs-based tests approved by the US Food and Drug Administration (FDA) for HIV testing (Table 1) demonstrated equal or higher sensitivity in a shorter time, in comparison with conventional ELISA [131].

Briefly, the performance study with LFA AuNP-based POC tests presented a sensitivity higher than 95% and a specificity higher than 99% in approximately 6300 whole-blood plasma samples [131]. Some of these companies, such as Alere, OraSure Tecnologies and Trinity Biotech applied similar strategies to other relevant targets, such as influenza, malaria, ebola and others (more information may be found at the companies’ sites or on demand). Multinational companies in the health-care and pharmaceutical sector, like Roche Diagnostics, are also developing new LFA POC-based strategies for diagnosis of human health conditions like heart failure, targeting cardiac biomarkers (i.e., myoglobin, troponin, and others). This first AuNP-based LFA screening platform presented by Roche Diagnostics is an AuNP-labeled LFA POC test without pre-sample treatment requirements, delivering accurate results in 8–12 min [131,132,133].

The Verigene^®^ System, introduced by Nanosphere, Inc (now part of Luminex Corporation, Austin, TX, United States) paves the way of future POC AuNP stand-alone technologies. Powered by NanoGrid Technology that consists of specific and patent chemistry for the functionalization of AuNPs, the system is capable of identifying and quantifying relevant nucleic acid sequences in biological samples with a fully automated platform. In 2009/2010 the Verigene^®^ System presented two FDA-approved POC tests for the identification of single-nucleotide polymorphisms associated with coagulation factors *F5* (1691G > A), *F2* (20210G > A) and *MTHFR* (677C > T) [134] and warfarin metabolism (CYP2C9*2, CYP2C9*3, *VKORC1*) [135]. Both of these tests presented similar results with no miss-calls obtained for all SNPs. The Verigene^®^ System is also applied in a wide range of pathogens present in the bloodstream, gastrointestinal and respiratory tract. The need for power supply, low portability and high level of instrumentation still hampers the full implementation in the field. As such, this platform only has been fully operational in developed and high-income countries.

## 5. Conclusions and Outlook

Nanomaterials, such as AuNPs, present a vast and unique set of properties useful for optimizing POC assays. In fact, POC assays incorporating nanotechnology, and in particular AuNPs, have been changing the way we perceive the next generation of molecular diagnostics. Significant advances in microfluidics and microfabrication will assist the growth of portable devices capable of delivering sensitive diagnostics at lower costs. Nanomaterials can greatly contribute to the development of on-site platforms capable of performing all analytical steps, from sample purification to analytical processing and data handling. However, international standardization and regulatory guidelines are required for the development and characterization of nanomaterials [136]. The development of new technology must be intensively validated against the gold standard technique following proper guidelines, suitable to be verified and certified by regulatory entities. This is the utmost relevance since often the novel detection platforms clearly outperform current techniques and validation is far from trivial. Performance control of POC tests may be highly variable according to local setting (i.e., temperature, humidity, etc.), thus setting specific sets of ranges depending on environmental conditions [137].

Progress in the development of nanotechnology POC diagnostic tests with real clinical value to be used at point of need is not far away. The impact of these novel nanomaterial-based platforms will be strongly felt in low-income regions, where development of these POC diagnostic tests will greatly contribute to the improvement of health conditions.

## Figures and Tables

**Table 1:** LFA AuNP-based systems on the market.

Company	Product Name ^1^	Principle of Detection	Sensitivity and Specificity ^2^	Results Time
Alere, Inc. (Waltham, MA, USA)	Clearview^®^ HIV 1/2 STAT-PAK	Gold-labeled lateral-flow immunoassay	99.7%/99.9%	10–15 min
	Clearview^®^ COMPLETE HIV 1/2		99.7%/99.9%	15 min
OraSure Technologies, Inc. (Bethlehem, PA, USA)	OraQuick ADVANCE^®^ HIV-1/2		98.7%/99.9%	20–40 min
MedMira, Inc. (Halifax, Nova Scotia, Canada)	Reveal^®^ G3 HIV-1		99.8%/99.1%	<3 min
Trinity Biotech Plc (Bray, Republic of Ireland)	Uni-Gold Recombigen™ HIV-1		100%/99.8%	10 min

^1^ All of these POC test are Food and Drug Administration (FDA)-approved for HIV testing; ^2^ Values can vary depending on biological sample plot for analysis (whole blood, serum, plasma or oral fluid).
